# Antiviral Activity of Umifenovir In Vitro against a Broad Spectrum of Coronaviruses, Including the Novel SARS-CoV-2 Virus

**DOI:** 10.3390/v13081665

**Published:** 2021-08-23

**Authors:** Irina Leneva, Nadezhda Kartashova, Artem Poromov, Anastasiia Gracheva, Ekaterina Korchevaya, Ekaterina Glubokova, Olga Borisova, Anna Shtro, Svetlana Loginova, Veronika Shchukina, Ravil Khamitov, Evgeny Faizuloev

**Affiliations:** 1 Mechnikov Research Institute of Vaccines and Sera, 105064 Moscow, Russia; nadezdakartasova10571@gmail.com (N.K.); poromov@insmech.ru (A.P.); anastasiia.gracheva.95@mail.ru (A.G.); c.korchevaya@gmail.com (E.K.); eaglubokova@yandex.ru (E.G.); olga.v.borisova@gmail.com (O.B.); kriatal49@mil.ru (S.L.); veronika6717@mil.ru (V.S.); faizuloev@mail.ru (E.F.); 2 Smorodintsev Research Institute of Influenza, 197376 Saint-Petersburg, Russia; anna.shtro@influenza.spb.ru; 3 International Biotechnology Center IBC “GENERIUM”, Volginsky Village, Petushinsky District, 601125 Vladimir, Russia; khamitov@ibsgenerium.ru

**Keywords:** coronaviruses, SARS-CoV, SARS-CoV-2, umifenovir, antiviral activity

## Abstract

An escalating pandemic of the novel SARS-CoV-2 virus is impacting global health, and effective antivirals are needed. Umifenovir (Arbidol) is an indole-derivative molecule, licensed in Russia and China for prophylaxis and treatment of influenza and other respiratory viral infections. It has been shown that umifenovir has broad spectrum activity against different viruses. We evaluated the sensitivity of different coronaviruses, including the novel SARS-CoV-2 virus, to umifenovir using in vitro assays. Using a plaque assay, we revealed an antiviral effect of umifenovir against seasonal HCoV-229E and HCoV-OC43 coronaviruses in Vero E6 cells, with estimated 50% effective concentrations (EC50) of 10.0 ± 0.5 µM and 9.0 ± 0.4 µM, respectively. Umifenovir at 90 µM significantly suppressed plaque formation in CMK-AH-1 cells infected with SARS-CoV. Umifenovir also inhibited the replication of SARS-CoV-2 virus, with EC50 values ranging from 15.37 ± 3.6 to 28.0 ± 1.0 µM. In addition, 21–36 µM of umifenovir significantly suppressed SARS-CoV-2 virus titers (≥2 log TCID50/mL) in the first 24 h after infection. Repurposing of antiviral drugs is very helpful in fighting COVID-19. A safe, pan-antiviral drug such as umifenovir could be extremely beneficial in combating the early stages of a viral pandemic.

## 1. Introduction

Coronaviruses are enveloped RNA viruses of the Coronaviridae family that cause acute respiratory illness. The Coronaviridae family constitutes the subfamily Orthocoronavirinae, classified into four CoV genera: Alphacoronavirus (alpha-CoV); Betacoronavirus (beta-CoV); Deltacoronavirus (delta-CoV); and Gammacoronavirus (gamma-CoV). Alphacoronaviruses include species of human coronavirus 229E and human coronavirus NL63, which affect human health. Betacoronavirus genera are divided into four lineages (subgroups A,B,C,D). Subgroup A includes Betacoronavirus 1 (human coronavirus OC43) and human coronavirus HKU1 [1,2,3,4]. In humans, the most common seasonal respiratory infections can be caused by two species: HCoV-229E (α-Coronavirus) and HCoV-OC43 (β Coronavirus). HCoV-229E and HCoV-OC43 were identified in the mid-1960s and are known to cause common cold-like and mild upper respiratory infections in immunocompetent individuals. Lower respiratory infections may occur in older or immunocompromised people [3,4]. Betacoronavirus subgroup B includes Severe Acute Respiratory Syndrome-related coronaviruses (SARS-CoV, SARS-CoV-2) [2,5]. Subgroup C includes the Middle East Respiratory Syndrome-related coronavirus as a human pathogen [2,6]. SARS-CoV infections began from 2002 to 2004 [5]. Middle East Respiratory Syndrome-related coronavirus (MERS-CoV) was identified in 2012 [6]. SARS-CoV-2 is a novel coronaviral strain that causes ‘coronavirus disease 2019’ (COVID-19); it has since been declared a pandemic by the WHO (March 2020) [2,7,8]. 

Clinical features of COVID-19 vary from mild acute respiratory viral infections to severe respiratory distress syndromes [7,8,9]. Mild to moderate forms of COVID-19 can be effectively treated with antivirals, but severe forms of COVID-19 (characterized by viral pneumonia or pulmonary immunopathology) require different approaches to treatment or drug combinations. There are no currently-licensed drugs developed using modern drug discovery approaches. An efficient approach to drug discovery is to evaluate whether existing approved drugs can be efficient against SARS-CoV-2. Broad-spectrum antiviral compounds previously reported to show effectiveness against different acute respiratory viral infections (ARVI) may be effective against SARS-CoV-2. 

Umifenovir (trade name Arbidol; ethyl-6-bromo-4-[(dimethylamino)methyl]-5-hydroxy-1-methyl-2-[(phenylthio)methyl]-indole-3-carboxylate hydrochloride monohydrate) is an oral, broad-spectrum antiviral with demonstrated activity against different viruses [10]. Numerous studies have shown that umifenovir blocks viral fusion [11,12,13,14]. It has received licensing for the treatment and prophylaxis of acute respiratory viral infections (including influenza A, B) in Russia (1993) and China (2006). It is safe and well tolerated in humans. As a broad-spectrum antiviral agent, it is active against numerous viruses, including influenza; hepatitis B and C; gastroenteritis agents; ebola; herpes; and some arthropod-borne flaviviruses, including Zika, West Nile, and tick-borne encephalitis virus [14,15,16,17,18].

In silico studies have demonstrated that umifenovir inhibits SARS-CoV-2 adhesion to host cell ACE2 receptors by impeding trimerization of spike glycoprotein [19,20]. Umifenovir has been used in Russia, China, and Iran to treat patients with COVID-19, either alone or in combination with other antiviral drugs; symptom-targeting approaches; or traditional medicine. Retrospective analyses of clinical data from patients with COVID-19 in China showed that umifenovir treatment was associated with less in-hospital death, faster viral elimination, and a lower frequency of severe courses and complications [21,22]. On the other hand, retrospective studies show umifenovir has potential for post-exposure prophylaxis among contacts and health care workers [23,24]. The National Health Commission of the People's Republic of China cites umifenovir (Chinese literation ‘Abidol’) in the *Diagnosis and Treatment Protocol for COVID-19 Patients (Tentative 8th Edition)* for treatment of non-severe COVID-19 patients [25].

According to the 11th edition of the *Interim Guidelines for the Prevention, Diagnosis and Treatment of Novel Coronavirus Infection (COVID-19)* issued by the Russian Ministry of Health, options for antiviral therapy include umifenovir [26]. The choice of antivirals for COVID-19 therapy is based on clinical practice, experimental data, and clinical trial results. Here, we report the activity of umifenovir against a spectrum of coronaviruses, including the novel SARS-CoV-2 agent, in vitro.

## 2. Materials and Methods

### 2.1. Cells and Viruses

Vero E6 cells (ATTC C1008) and Vero CCL81 cells were used to study antiviral activity against seasonal coronaviruses (HCoV-OC43, HCoV-229E) and SARS-CoV-2 virus (‘Dubrovka’ strain), respectively. GMK-AH-1(D) (African green monkey epithelial-like) cells (RRID:CVCL_L878) were used to study the antiviral activity against SARS-CoV virus. Cell lines were grown at 37 °C, with 5% CO_2_, in DMEM (Pan Eko, Moscow, Russia) supplemented with 5% heat-inactivated fetal bovine serum (FBS, Invitrogen, Carlsbad, CA, USA); 4.5 g/L of glucose (Sigma-Aldrich, St. Louis, MO, USA); 300 µg mL^−1^ of L-glutamine (Pan Eko, Russia); and 40 μg/mL gentamicin (Sigma-Aldrich, St. Louis, MO, USA).

Seasonal human coronavirus HCoV-229E was obtained from the ATTC (VR-740). The seasonal human coronavirus HCoV-OC43 was a clinical isolate that was prepared after serial passages in Vero E6 cells in ‘infection media’ (DMEM supplemented with 1% heat-inactivated FBS, 4.5 g/L of glucose, 300 µg/mL of L-glutamine, and 40 μg/mL gentamicin). SARS-CoV virus (RU2263144C2) was a clinical isolate from patient that was prepared after serial passages in Vero E6 cells in ‘infection media’. SARS-CoV-2 virus (‘Dubrovka’ strain, GenBank: MW161041.1) was isolated from the nasopharynx aspirate and throat swab of the confirmed COVID-19 patients in Vero CCL81 cells. Viral stocks (3.2 × 10^7^ TCID50/mL) were prepared after serial passages in Vero CCL81 cells using ‘infection media’ (detailed above). For all viruses, stocks were kept as aliquots at −80 °C. 

### 2.2. Compounds

Umifenovir (Arbidol^®^, OTC Pharma, Moscow, Russia) was dissolved as a 10 mM stock in 96% ethanol at 37 °C for 10 min, followed by dilution in sterile, distilled water. For each experiment, a freshly-made stock was used. Hydroxychloroquine (CAS: 118-42-3) and ribavirin (CAS: 36791-04-5) were dissolved in distilled water as 5 mM stocks and stored as aliquots at −20 °C. 

### 2.3. Determination of Cell Viability

The cytotoxicity of umifenovir was assessed by MTT assay. Briefly, cells (Vero CCL81, Vero E6, GMK-AH-1(D)) at a density of 2 × 10^4^ cells/well were seeded into flat-bottom, 96-well microtiter plates and incubated until formation of a confluent monolayer (37 °C, 5% CO_2_). A range of concentrations (1.8 to 180 µM umifenovir) was prepared using cell culture medium and added to plates in quadruplicate (200 μL). After 72 h, the treatments were removed, and 40 μL of MTT reagent (5 µg m/L) was added to each well and incubated for a further 2 h. Media was then removed, and 100 μL of DMSO solution was added to the wells. Finally, plates were read at 550 nm by a microplate reader (Varioskan Flash, Thermo Scientific, Waltham, MA, USA). The percentage cell viability was calculated using the following formula: Cell viability (%) = ((OD of untreated cells − treated cells)/(OD of untreated cells)) × 100. The 50% cytotoxicity concentration (CC50) was defined as the cytotoxic concentration of each compound that reduced the absorbance of treated cells to 50% when compared with that of untreated cells. The 90% survival cell value (CC10) was defined as the cytotoxic concentration of each compound that decreased cell viability by 10% in comparison to control cells.

### 2.4. Antiviral Activity Assessment

#### 2.4.1. Activity against Seasonal HCoV-OC43 and HCoV-229E Coronaviruses

Confluent Vero E6 cell monolayers were incubated in medium with or without compounds, at a range of concentrations from 180 to 5.4 µM, in 6-well plates for 1 h (37 °C, 5% CO_2_). Viruses (MOI = 0.01) were added to the cells and incubated for 2 h (37 °C, 5% CO_2_). After washing, cells were covered with medium containing 0.9% agar (Merck, Burlington, MA, USA), with and without compound; incubated for 8 days (37 °C, 5% CO_2_); and fixed and stained with 5% crystal violet solution containing 10% buffered formalin. The resulting plaques were then counted. The percentage decrease or increase in plaques was calculated using the following formula: Percentage plaque reduction (%) = ((plaque number of treated cells)/(plaque number of untreated cells)) × 100. The EC50 was defined as the effective drug concentration that reduced viral titers (in culture supernatants of infected cells) to 50%, when compared with those of virus controls. 

#### 2.4.2. Activity against SARS-CoV

GMK-AH-1(D) cells were seeded into 6-well plates. The next day, cells were infected with SARS-CoV (0.003 PFU/cell). Umifenovir (90 µM) was added to the GMK-AH-1(D) cells 4 h before, immediately after, and 2 h after infection. The positive control (ribavirin) and the negative control (no drug) were spotted on each plate. After incubation for 72 h (37 °C, 5% CO_2_ with humidification), culture supernatants were collected. Supernatant titers were determined by plaque assay, using Vero E6 cells, as described above. 

#### 2.4.3. Activity against SARS-CoV-2

Antiviral activity against the SARS-CoV-2 virus strain Dubrovka was determined using three different assays: MTT cell viability; viral replication in cell-ELISA; and inhibition of viral titer. In all assays, confluent monolayers of Vero CCL81 cells (2 × 10^4^ cells/well) in flat-bottom, 96-well microtiter plates were pre-incubated with 100 μL of maintenance medium, without and with different non-toxic umifenovir concentrations (from 1.8 to 54 µM), in quadruplicate for 2 h at 37 °C. The ‘maintenance medium’ featured the same composition as the ‘growth medium’, except for the concentration of FCS (1%). ‘Virus controls’ (infected, but untreated) and ‘cell controls’ (uninfected, untreated cells) were included on each plate prepared throughout the experiment. After 2 h of incubation, different doses of virus (from 0.0005 to 0.001 MOI, in 100 μL) were added to wells, except ‘cell control’ wells, and cells were incubated for a defined period (37 °C, 5% CO_2_ with humidification). 

In the first assay, after incubation for the indicated time, the medium was removed, and 40 μL of MTT reagent (5 µg/mL) was added to each well and incubated for a further 2 h. Then, the inoculum was discarded and 100 μL of DMSO solution was added to the wells. Finally, the plates were read at 550 nm by a microplate reader (Varioskan Flash, Thermo Scientific, Waltham, MA, USA). The EC50 was defined as the inhibitory concentration of compound that reduced the absorbance of treated infected cells to 50% when compared with that of cell controls. 

For performing the cell-ELISA assay, after incubation for 24 or 48 h, medium was removed, and cells were fixed by adding 50 μL of cold 80% acetone in PBS at RT for 20 min. SARS-CoV-2 nucleoprotein expression was measured by ELISA. Briefly, fixed cells were washed with 0.05% Tween-20 PBS and incubated with 100 μL of ELISA buffer (PBS with 1% BSA, 0.1% Tween-20) for 1 h. Cells were then incubated with 500 ng/mL of mAb to SARS-CoV-2 nucleoprotein (HyTest, RF) in ELISA buffer at 37 °C for 1 h. After washing, cells were incubated with rabbit anti-mouse-IgG peroxidase conjugate (Sigma-Aldrich, A9044, 1:10,000 dilution) at 37 °C for 45 min. After further washing, substrate solution was added (0.01% (*w*/*v*) 3.3´, 5.5´-tetramethylbenzidine/citrate buffer, pH 4.0, with 0.03% (*v*/*v*) H_2_O_2_). Following 15 min of development, reactions were stopping with 0.5 M H_2_SO_4_. Optical densities (O.D.) were measured at 450 nm, with subtraction of densities at 620 nm, the reference wavelength (Infinite F50, Tecan Microplate Reader). The percent inhibition of viral replication (by antivirals) was calculated, after correcting for background (cell control) values, as follows: percent inhibition = 100 × (1 − (OD) treated sample/(OD) ‘virus control’ sample). EC50 values (the concentration of compound required to inhibit viral replication by 50%) were determined by plotting the percent inhibition of viral replication as a function of compound concentration. In the virus titration assay, after incubation for 24 h and 48 h, culture supernatants were collected to quantify viral loads by titration using the tissue culture infectious dose 50 (TCID50) method, according to the Ramakrishnan formula [27].

#### 2.4.4. Pre- and Post-Exposure Antiviral Activity against SARS-CoV-2

In pre-exposure experiments, confluent monolayers of Vero CCL81 cells (2 × 10^4^ cells/well) in flat-bottom, 96-well microtiter plates were pre-incubated for 24 h and 2 h with 100 μL of maintenance medium, without and with umifenovir (31.5 µM), in quadruplicates at 37 °C. After incubation for the defined times, virus (0.001 MOI) was added to the wells (except ‘cell control’ wells), and cells were incubated for 5 days (37 °C, 5% CO_2_ with humidification).

In post-exposure experiments, confluent monolayers of Vero CCL81 cells (2 × 10^4^ cells/well) in flat-bottom, 96-well microtiter plates were infected with 100 μL of (0.001 MOI) viral suspensions. Umifenovir (31.5 µM in 100 μL of maintenance medium) was then added for the defined times, in quadruplicate, to the wells. The ‘virus control’ (virus + DMEM) and the ‘cell control’ (uninfected cells in DMEM) were also included in this experiment. In both the pre-and post-exposure experiments, following incubation of the plates for 5 days after infection (37 °C, 5% CO_2_ with humidification), media were discarded from the wells, and the MTT cell viability assay was further carried out, as described above. 

#### 2.4.5. Statistical Analysis

All data represent three independent experiments. Statistical analyses were performed using R-Studio software (version 1.0.143). Analysis of dose–response data was made in the package ‘drc’, ver. 3.0-1. The three-parameter log-logistic (LL.3) function was used. Estimating effective doses, namely 50% cytotoxic concentration (CC50) and 50% effective concentration (EC50), was conducted with asymptotic-based confidence intervals. Estimating 50% effective concentration (EC50) in cell-ELISA was done in Excel. A Kruskal–Wallis test was used to determine differences between two or more groups. Data were expressed as mean ± standard deviation (SD). A *p*-value of <0.05 was considered statically significant.

## 3. Results

### 3.1. Cytotoxic Effect of Umifenovir in Different Cells

**Figure 1:**
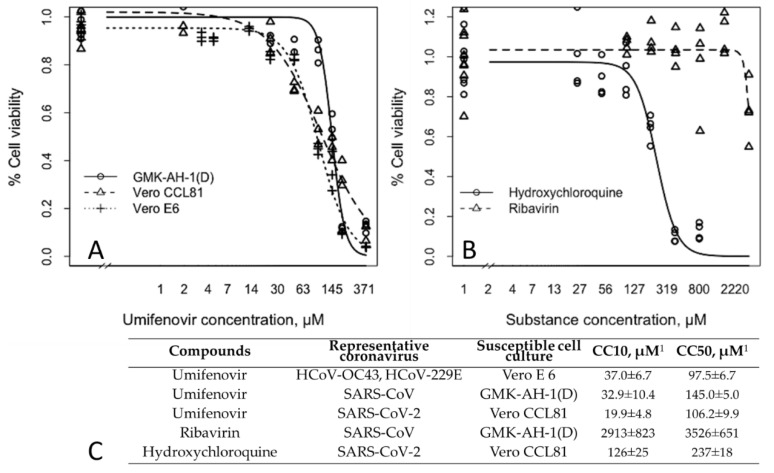
Cytotoxicity of antivirals in different cell lines. Vero E6, GMK-AH-1(D), and Vero CCL81 cells were seeded into 96-well microtiter plates and incubated until formation of a confluent monolayer. Umifenovir (**A**), ribavirin, and hydroxychloroquine (**B**) were added at various concentrations. The impact of treatment on cell viability was assessed by MTT assay after 72 h incubation. CC10 and CC50 values were generated and represented as mean ± SD (**C**). The three-parameter log-logistic (LL.3) function was used. ^1^ CC10 and CC50 were determined from three independent experiments.

A cytotoxicity assay was performed to clarify the non-toxic concentration of umifenovir in different cells that we used in our studies. The CC50 values for umifenovir were 97.5 ± 6.7 µM in Vero E6 cells, and 106.2 ± 9.9 µM in Vero CCL81 cells. Umifenovir was less toxic in GMK-AH-1(D) cells, with a CC50 value of 145.0 ± 5.0 µM (Figure 1A,C). CC10 survival values of umifenovir corresponded to CC50 values in cells studied. The CC50 values of umifenovir here were consistent with previously described values [28]. Ribavirin and hydroxychloroquine were less toxic than umifenovir in GMK-AH-1(D) and Vero CCL81 cells, respectively (Figure 1B,C). 

### 3.2. Antiviral Activity of Umifenovir against Seasonal Coronaviruses

**Figure 2:**
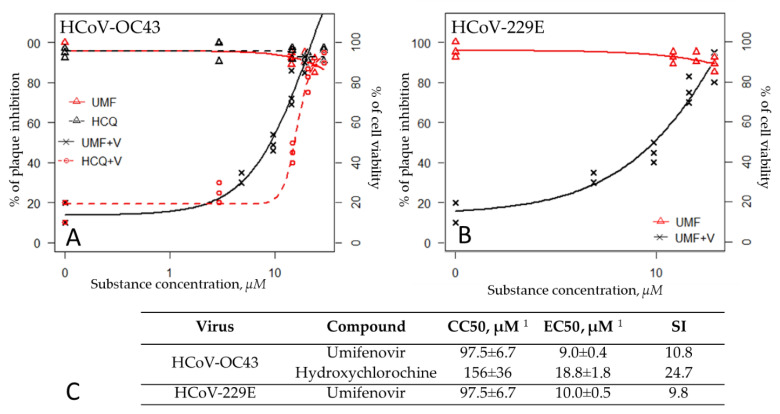
Antiviral activity of umifenovir against seasonal coronaviruses. Umifenovir (UMF) and hydroxychloroquine (HCQ) were added to Vero E6 cells at each concentration. The rate of cell viability (cytotoxicity control) was measured after 72 h using the MTT assay. HCoV-OC43 and HCoV-229E virus growth was determined by plaque assay 8 days after infection in the presence of various concentrations of drugs. (**A**) Percent inhibition of HCoV-OC43 by umifenovir (UMF+V) and hydroxychloroquine (HCQ+V) and cytotoxicity (UMF, HCQ) in Vero E6. (**B**) Percent inhibition of HCoV-229E by umifenovir (UMF+V) and cytotoxicity (UMF) in Vero E6. (**C**) The CC50 and EC_50_ values are represented as mean ± SD, and the three-parameter log-logistic (LL.3) function was used. Selectivity index (SI) was calculated as a ratio of a drug’s CC50 and EC50 values (SI = CC50/EC50). ^1^ CC50 and EC50 were determined from three independent experiments.

The antiviral effect of umifenovir against both seasonal coronavirus serotypes (HCoV-OC43 and HCoV-229E) was evaluated using plaque inhibition assays in Vero E6 cells. In cells infected at a 0.01 MOI, umifenovir inhibited replication of both viruses in a dose-depended manner. The EC50 of umifenovir was 9.0 ± 0.4 µM against HCoV-OC43 and 10.0 ± 0.5 µM against HCoV-229E (Figure 2). The SIs were similar for both viruses (10.8 and 9.8 for HCoV-OC43 and HCoV-229E, respectively) (Figure 2C). 

### 3.3. Activity against SARS-CoV

Since umifenovir has shown antiviral effects against seasonal coronaviruses and other enveloped respiratory viruses [10,13,14,28], we expected to see a similar effect with the related, highly pathogenic SARS-CoV coronavirus. To this end, we evaluated the effect of umifenovir on SARS-CoV plaque formation (GMK-AH-1(D) cells), depending on timing of drug addition. 

**Figure 3:**
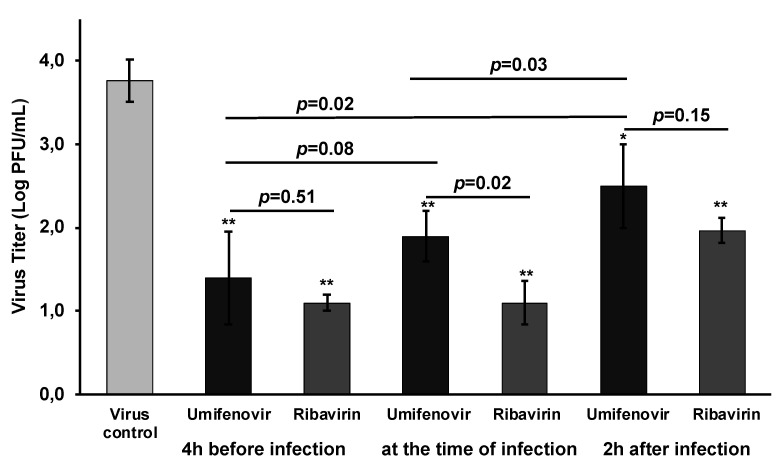
Effects of pre- and post-exposure with umifenovir on SARS-CoV viral titers. SARS-CoV-infected GMK-AH-1(D) cells were incubated with umifenovir (90 µM) 4 h before, immediately after, and 2 h after infection. Viral titers (log PFU/mL) are shown as mean ± SD. Differences between virus control group and treated groups: * *p* < 0.05, ** *p* < 0.01. Kruskal–Wallis test.

Umifenovir at a dose of 90 µM and ribavirin at a dose 50 mg/mL (used as a control) statistically significantly suppressed SARS-CoV plaque formation at any addition regime. However, inhibition by umifenovir was higher when umifenovir was added to cells before or simultaneously with infection than it was added after infection (Figure 3). In our experiments, the antiviral effects of ribavirin and umifenovir on viral titers did not differ significantly when drugs were added 4 h before and 2 h after infection, but ribavirin was slightly superior when drugs were added immediately after infection (Figure 3). This fact is unclear and future studies are needed. 

### 3.4. Activity against SARS-CoV-2

**Figure 4:**
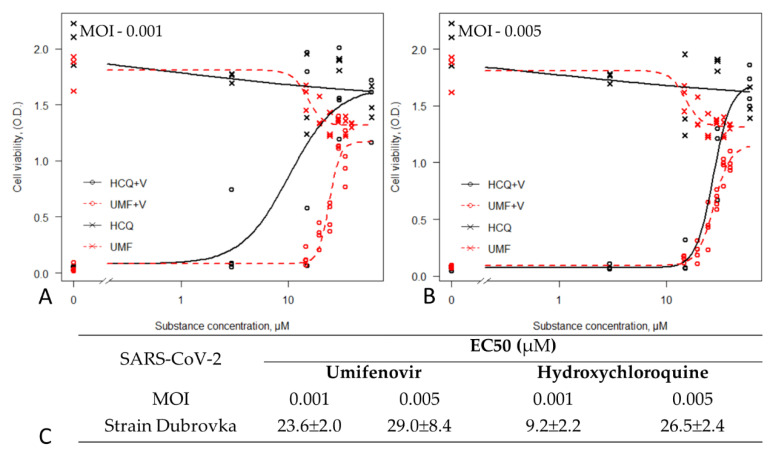
Antiviral activity of umifenovir against SARS-CoV-2 virus. Umifenovir (UMF) and hydroxychloroquine (HCQ) were added to Vero CCL81 cells at various concentrations. The cellular cytotoxicity test of Vero CCL81 cells was included in the experiment, and the resulting curves are shown (UMF, HCQ). After 2 h of incubation, virus at dose of 0.001 (**A**) and 0.005 MOI (**B**) was added to wells, except ‘cell control’ wells, and cells were incubated at 37 °C in a humidified 5% CO_2_ atmosphere for 5 days. The CPE and rate of cell viability was measured using MTT assay. (**A**) Percent inhibition of SARS-CoV-2 (strain Dubrovka) by umifenovir (UMF+V) and hydroxychloroquine (HCQ+V) at MOI 0.001 and cytotoxicity (UMF, HCQ). (**B**) Percent inhibition of SARS-CoV-2 (strain Dubrovka) by umifenovir (UMF+V) and hydroxychloroquine (HCQ+V) at MOI 0,005) and cytotoxicity (UMF,HCQ). (**C**) The EC50 values are represented as mean ± SD; the three-parameter log-logistic (LL.3) function was used.

It has been shown that the SARS-CoV virus is closely related to the SARS-CoV-2 virus [2]. Firstly, we studied the effect of umifenovir on viral replication of SARS-CoV-2 clinical isolate (strain Dubrovka), at high and low multiplicities of infection. The antiviral effects of umifenovir against the clinically isolated SARS-CoV-2 strain were concentration-dependent, and stronger with increasing drug concentration. The EC50 (umifenovir) values for the strain Dubrovka at 0.001 and 0.005 MOI, were 23.6 ± 2.0 µM and 29.0 ± 8.4 µM, respectively (Figure 4A,C).We selected hydroxychloroquine as the positive control in our study, and the results showed that the EC50 values for hydroxychloroquine ranged from 9.2 ± 2.2 to 26.5 ± 2.4 (Figure 4B,C). These are consistent with previous results in Vero E6 cells (at MOIs of 0.2 to 0.25) [29,30,31].

**Figure 5:**
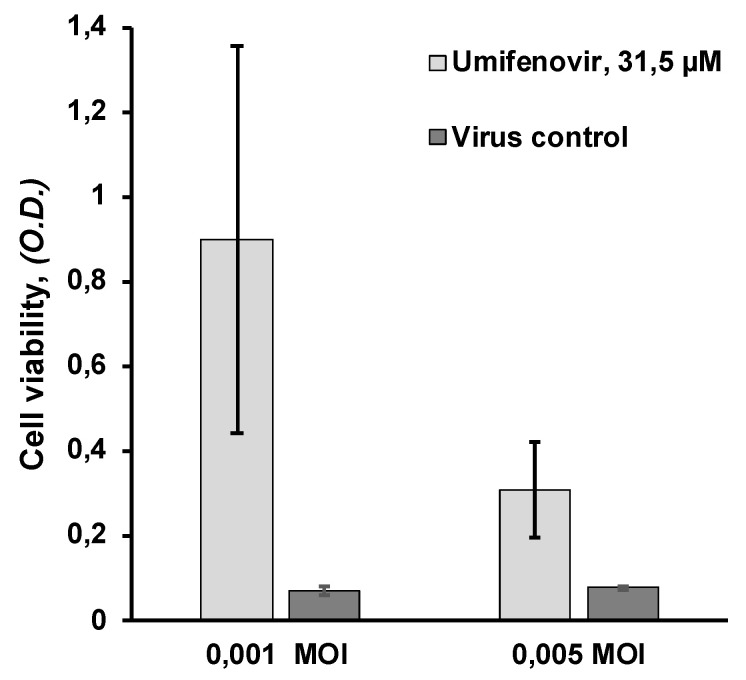
Effects of low and high multiplicity of infection on umifenovir antiviral activity. SARS-CoV-2 Dubrovka strain-infected Vero CCL81 cells were pre-treated with 31.5 µM umifenovir for 2 h at 0.001 and 0.005 MOI. After incubation for 5 days at 37 °C in a humidified 5% CO_2_ atmosphere, the cell viability was estimated by MTT assay. The optical density (OD) is shown as the mean ± SD. Data represent three replications. Cell control—1.59 ± 0.42 (*n* = 6), umifenovir control (without virus)—1.47 ± 0.54 (*n* = 3).

Further, the level of inhibition by umifenovir also depended on the multiplicity of infection and increased with decreasing viral dose. Umifenovir at 31.5 µM (close to its EC50) decreased virus-induced CPE much more significantly at a low dose of virus (0.001 MOI) than at a high dose of virus (0.005 MOI) (Figure 5).

**Figure 6:**
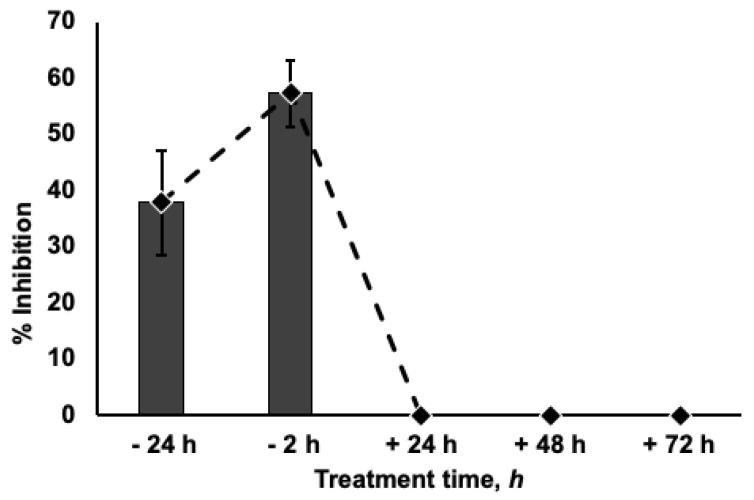
Effect of pre- and post-exposure treatment on antiviral activity of umifenovir. Umifenovir at dose 31.5 µM was added to Vero CCL81 cells. After incubation for 24 and 2 h, the virus (SARS-CoV-2 Dubrovka strain 0.001 MOI) was added. For post-exposure treatment Vero CCL81 cells were infected with the same dose of virus. Umifenovir at a concentration of 31.5 µM was then added 24, 48, or 96 h after infection.

Moreover, the antiviral effect of umifenovir depended on drug addition timing. Our experiments showed that umifenovir at 31.5 µM was most effective when it was added 2 h before infection. The level of inhibition decreased when the pre-incubation time was increased to 24 h. Addition of umifenovir after infection did not significantly affect viral yield (Figure 6). These results are in good agreement with previous studies of umifenovir activity against influenza viruses; analysis of umifenovir kinetics (MDCK cells) showed that its half-life is approximately 24 h, which explains reduced umifenovir effects under these conditions [32]. Our results about absence of inhibition (when umifenovir was added after infection) suggest that umifenovir was active during initial or early SARS-CoV-2 entry in the Vero CCL81 cells. These data are in line with numerous studies where umifenovir was reported to inhibit fusion between the viral envelope and target cell membranes [11,12,13].

Further, we studied the antiviral activity of umifenovir against SARS-CoV-2 virus using cell ELISA assays. In these experiments, we measured the direct effect of umifenovir on expression of SARS-CoV-2 viral protein before development of CPE in cells (24 and 48 h after infection). In this assay, umifenovir inhibited the viral expression of SARS-CoV-2 virus (strain Dubrovka), and the level of inhibition depended on the multiplicity of infection and the incubation time of infected cells. As with the MTT cell viability assay, inhibition in cell-ELISA assay increased with decreasing virus dose. In addition, the EC50 values were higher at 48 h, than at 24 h, after cell infection (Table 1, Supplementary Figure S1).

**Figure 7:**
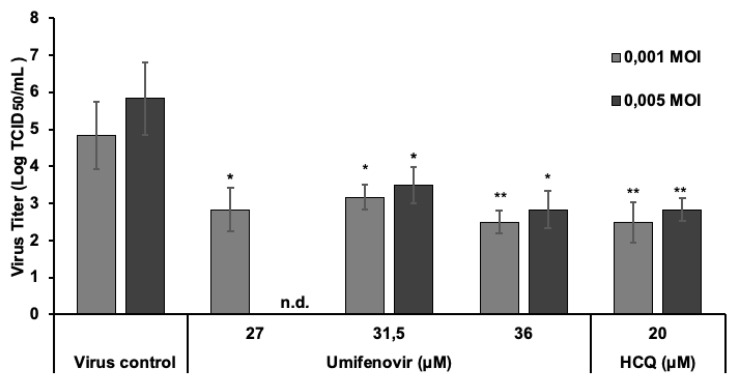
Effect of umifenovir and hydroxychloroquine (HCQ) on viral titers in SARS-CoV-2-infected Vero CCL81 cells at low and high multiplicities of infection. Vero CCL81 cells were pre-treated with drugs for 2 h, and virus at doses of 0.001 and 0.005 MOI was then added. After incubation or 24 h, the culture supernatants were collected to quantify viral loads by titration using the tissue culture infectious dose 50 (TCID50) method. The viral titers (log TCID50/mL) are shown as mean ± SD. Data represent three independent experiments. Differences between virus control group and treated groups: * *p* < 0.05, ** *p* < 0.01. Kruskal–Wallis test. n.d.—not determined.

In the next experiments, the effect of umifenovir in cells infected with SARS-CoV-2 was assessed by performing virus titrations. Preliminary studies of viral titer kinetics in SARS-CoV-2-infected cells showed that the highest titer of virus was reached at 24 h after infection, remaining at the same level for a further 84 h (Supplementary Figure S2). Therefore, we evaluated the effect of umifenovir 24 h after infection. For the study, we chose umifenovir concentrations close to the EC50 values determined using the MTT cell viability and cell-ELISA assays. Under the conditions tested, SARS-CoV-2 titers significantly decreased in supernatants of cells pretreated with different umifenovir concentrations at both multiplicities of infection. The level of virus titer reduction was highest at umifenovir concentration of 36 µM, being 2.33 log TCID50/mL and 3.0 log TCID50/mL at 0.001 and 0.005 MOI, respectively. These values were comparable with those obtained with hydroxychloroquine (2.33 to 3.0 log TCID50/mL), used as a positive control (Figure 7). 

## 4. Discussion

Pandemics of new respiratory infections will always occur in the absence of prophylaxis and treatment. Identifying effective, affordable antiviral drugs with limited side effects, which are available immediately, is urgently needed. One of the efficient approaches to drug discovery is drug repurposing, which consists of evaluating whether existing, approved drugs can be effective against SARS-CoV-2. Umifenovir is a broad-spectrum antiviral that possesses activity against numerous viruses [13,14]. Here, we report data on the antiviral activity of umifenovir against a spectrum of coronaviruses, including the novel SARS-CoV-2 virus. Our study showed effective in vitro antiviral activity against the analyzed coronaviruses, including SARS-CoV-2. The EC50 values in Vero CCL81 cells ranged from 9.0 ± 0.4 µM to 46 ± 9.6 µM, depending on the coronavirus strain, assay, and timing of drug addition. It is known that antiviral effect of some antivirals against SARS-CoV-2 (HCQ, cathepsin L inhibitor E-64d and TMPRSS2 (transmembrane serine proteinase 2) inhibitor camostat mesylate) is cell type dependent and associated with levels of expression of ACE2 and transmembrane serine proteinase 2 (TMPRSS2). Vero E6 cells express high levels of ACE2 on the apical membrane domain but minimal levels of transmembrane serine proteinase 2 (TMPRSS2), the host serine protease that cleaves viral spike protein [33,34,35]. Taking into consideration that mechanism of action of umifenovir is associated with early stage of viral replication it will be interesting to study umifenovir in other types of cells. The study of antiviral activity of umifenovir in a human lung epithelial cell line Calu-3 with endogenous expression of both ACE2 and TMPRSS2 [35] is planned.

At present there are no reliable data on the mechanism of action of umifenovir against SARS-CoV-2 virus. It was shown by atomistic simulations that umifenovir inserted into a spike protein of SARS-CoV-2 virus bonded to the RBD/ACE2 interface and induced in it structural rigidity, blocking virus fusion into the cell [19,20]. A similar antiviral mechanism was shown for influenza virus. Analysis of hemagglutinin and umifenovir crystal structures showed that umifenovir binds in a hydrophobic cavity in the HA and stabilizes the prefusion conformation of HA that inhibits the large conformational rearrangements associated with membrane fusion in the low pH of the endosome [11,12]. Further and more detailed studies of the mechanism of action of umifenovir against SARS-CoV-2 virus are needed.

Our results showed that umifenovir has a rather poor selective index (SI) in cell cultures. However, other antiviral drugs with low SI, such as amantadine, rimantadine, and azidotimidine, have been shown to be active and have widespread clinical application. It should be also noted that in vitro data do not always translate into in vivo, and the cytotoxicity in cell culture may not reflect the toxicity of the drug in a host. In our experiments, hydroxychloroquine and ribavirin were less toxic in cell cultures than umifenovir; however, it is known that both drugs have serious side effects. On the contrary, clinical trials conducted in more than 30,000 patients and the experience of using umifenovir for 25 years in Russia has shown that umifenovir has been well tolerated and safe, and no serious adverse effects have been revealed. 

Moreover, the pharmacokinetics profile, such as maximal concentration (Cmax), is also very important for predicting efficacy. In humans, Cmax of umifenovir was 4.1 μM after single oral administration of 800 mg of umifenovir (daily dose) [36]. It is believed that if the Cmax achieves EC90, the drug is very likely to be effective, while if the Cmax achieves EC50, the drug is possibly effective in vivo. In most of our experiments, the EC50 values were comparable or higher than the achievable maximum plasma concentrations (Cmax) reported for umifenovir after oral administration of the approved human dose. However, it was shown in animals that umifenovir has a high tropism for lung tissue and is able to accumulate in the lungs [37], the main target organ for SARS-CoV-2 and other respiratory virus therapies. Therefore, a deeper understanding of the pharmacokinetics of umifenovir in cells and hosts will be needed.

Further, experimental and clinical studies have revealed that, in addition to direct antiviral umifenovir effects, its antiviral properties are also based on a capacity to induce interferon (IFN) antiviral responses [10], which are partially abrogated in the Vero cell line. This suggests that achieving sufficient therapeutic effects with umifenovir in the host may be possible at lower concentrations. This statement is confirmed by several retrospective analyses and clinical trials conducted in different countries (China and Iran) that demonstrated that umifenovir is effective in treatment and prophylaxis in patients with COVID-19. However, umifenovir efficiency against SARS-CoV-2 should be additionally studied in randomized clinical trials. To our knowledge, only two randomized clinical trial were performed in 56 and 100 patients with moderate COVID-19. Each trial included two groups of patients—the umifenovir treated group and the lopinavir/ritonavir or hydroxychloroquine treated group, respectively [38,39]. Despite the limited group size, umifenovir treatment accelerated the recovery process and the resolution of COVID-19 symptoms in these trials. Future randomized clinical trials in COVID-19 patients are necessary. 

Coronavirus genomic replication involves RNA-dependent RNA polymerase, and coronaviruses use RNA–RNA recombination for evolution [40,41]. Coronavirus biology inevitably will lead to the appearance of new pandemic strains; it is currently impossible to predict the moment of their development, genomic variability, or antigenic properties. As of today, several different SARS-CoV-2 variants have been identified. This underscores the necessity for prior research and development of antivirals for the prevention and treatment of a broad spectrum of coronaviruses. Our data show umifenovir’s antiviral activity against both seasonal (HCoV-OC43 and HCoV-229E) and highly pathogenic (SARS-CoV, SARS-CoV-2) coronaviruses. A major concern regarding the use of antiviral compounds to control infection is the emergence of drug-resistant variants. Umifenovir-resistant mutants of influenza A virus have been generated only in cell culture [11]. Currently, there is no evidence of naturally occurring resistance to umifenovir [42,43]. Clinical studies showed that a 5-day course of umifenovir therapy did not lead to the emergence of drug-resistant variants [43]. The totality of these data suggests that emergence of umifenovir-resistant coronaviruses through drug treatment is unlikely. 

It has been shown that umifenovir is active against all antigenic subtypes of human influenza A (including rimantadine- and oseltamivir-resistant strains) and influenza B viruses; influenza C virus; and avian influenza viruses possessing hemagglutinin types H5, H6, and H9 [14,16,42,44]. Umifenovir also exhibits wide-ranging, potent antiviral activity against a number of respiratory viruses including respiratory syncytial virus [45]; adenovirus [46]; parainfluenza type 5 and rhinovirus type 14 [28,46]; Coxsackie B3 virus; and rotavirus [46]. Thus, umifenovir has broad-spectrum antiviral activity against important respiratory viruses featuring similar symptoms. This makes it suitable as a drug without a diagnostic testing requirement before administration.

## 5. Conclusions

In conclusion, experimental data, clinical trials, and over 30 years of practical experience have demonstrated that umifenovir is effective in prophylaxis and treatment of acute respiratory viral infections, while being well tolerated and safe for humans. Based on experimental data on its antiviral activity against a broad range of coronaviruses (including SARS-CoV-2) and preliminary, encouraging data from ongoing clinical trials in COVID-19 patients, umifenovir likely has a role to play in multifaceted therapeutic approaches to COVID-19. However, future trials with sophisticated designs must be initiated. It is very important to assess the efficacy of umifenovir in different groups of patients with moderate to severe COVID-19 infection, including high-risk patients; and to determine the COVID-19 stage in which these treatments have the greatest benefit in terms of disease remission.

## Figures and Tables

**Table 1:** Antiviral activity of umifenovir against SARS-CoV-2, strain Dubrovka in cell-ELISA assay. Graphical data present in Supplementary Materials (Figure S1).

SARS-CoV-2, Strain Dubrovka	Time of Incubation			
	24 h		48 h	
moi	0.0025	0.001	0.0025	0.001
EC50 (µM) Umifenovir	11.2 ± 0.67	11.9 ± 1.1	46 ± 9.6	20 ± 7.4

## Data Availability

The data presented in this study are available on request from the corresponding author.

## Supplementary Materials

The following are available online at https://www.mdpi.com/article/10.3390/v13081665/s1, Figure S1: Kinetics of SARS-CoV-2 replication in Vero CCL81 cells (0.001 MOI). Figure S2: Antiviral activity of umifenovir against SARS-CoV-2, strain Dubrovka in cell-ELISA assay.
