# Supplementary material for: Antiviral Activity of Umifenovir In Vitro against a Broad Spectrum of Coronaviruses, Including the Novel SARS-CoV-2 Virus
Source: Viruses. 2021 Aug 23;13(8):1665. doi: 10.3390/v13081665 (PMC8402645; doi:10.3390/v13081665)
Supplement: Supplementary file 1 [file viruses-13-01665-s001.zip › viruses-1300794-SI.pdf]

viruses

MDPI

SUPPLEMENTARY MATERIALS

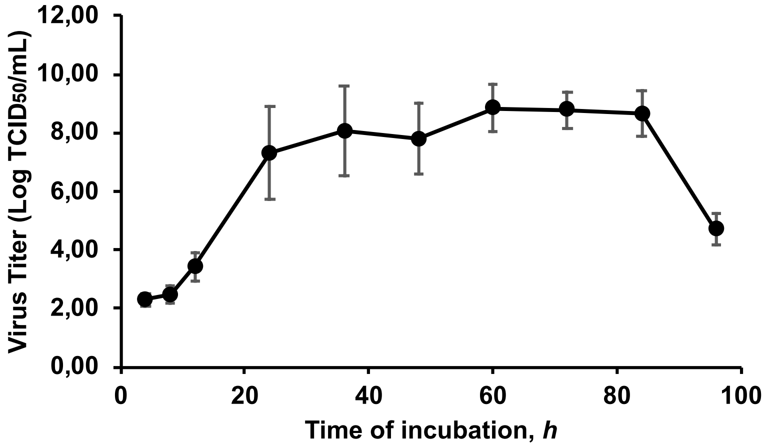

Figure S1. Kinetics of SARS-CoV-2 replication in Vero CCL81 cells (0.001 MOI). The cell monolayers were infected with 0.001 MOI and incubated for 24, 48, 60, 72 and 96 hours. After incubation for defined time the culture supernatants were collected and supernatant virus titers were determined using the tissue culture infectious dose 50 (TCID50) method. The virus titer (Log TCID50/mL) are shown as the mean±SD, data represent three independent experiments.

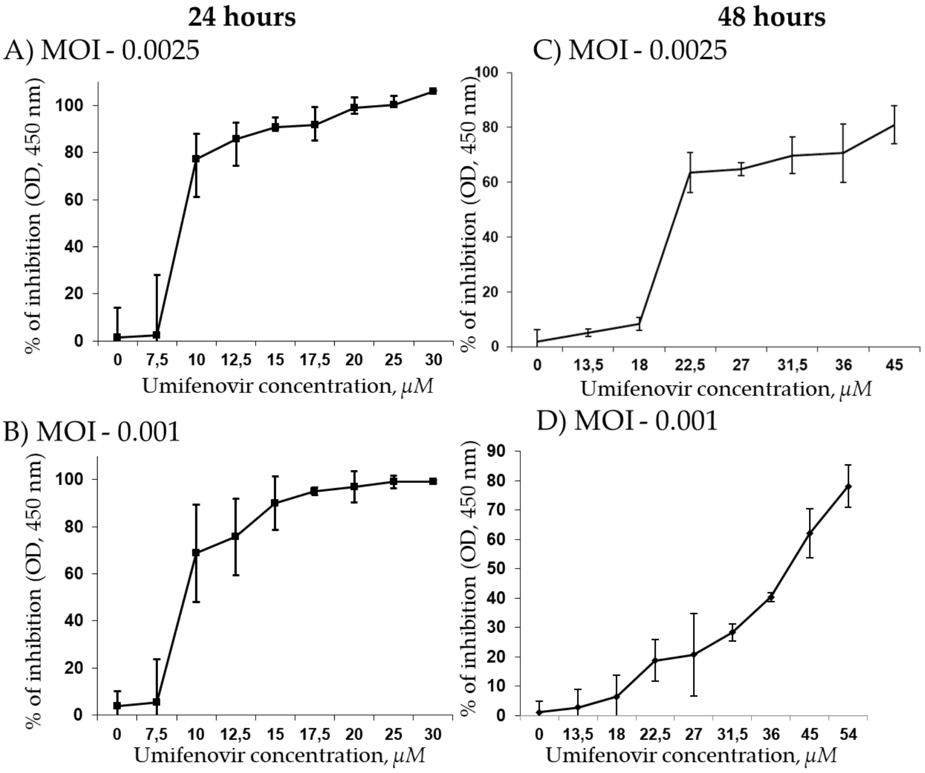

Figure S2. Antiviral activity of umifenovir against SARS-CoV-2, strain Dubrovka in cell-ELISA assay. Umifenovir was added to Vero CCL81 cells at a non-cytotoxic concentration range. After 2 hrs of incubation, virus at dose of 0.0025 (A,C) and 0.001 MOI (B,D) was added to wells, except 'cell control' wells, and cells were incubated at 37 °C in a humidified 5% CO2 atmosphere for 24 (A,B) or 48 (C,D) hrs. After fixation of cells with 80% acetone in PBS SARS-CoV-2 nucleoprotein expression was measured by ELISA as described in Methods. The EC50 values are represented as mean ± SD, the three-parameter log-logistic (LL.3) function was used.
